# Time-Resolved and Label-Free Evanescent Light-Scattering Microscopy for Mass Quantification of Protein Binding to Single Lipid Vesicles

**DOI:** 10.1021/acs.nanolett.1c00644

**Published:** 2021-05-18

**Authors:** Mattias Sjöberg, Mokhtar Mapar, Antonius Armanious, Vladimir P. Zhdanov, Björn Agnarsson, Fredrik Höök

**Affiliations:** † Division of Nano and Biophysics, Department of Physics, Chalmers University of Technology, Gothenburg 41296, Sweden; ‡ Boreskov Institute of Catalysis, Russian Academy of Sciences, Novosibirsk 630090, Russia

**Keywords:** surface-sensitive scattering microscopy, surface plasmon resonance, single nanoparticle analytics, protein adsorption kinetics

## Abstract

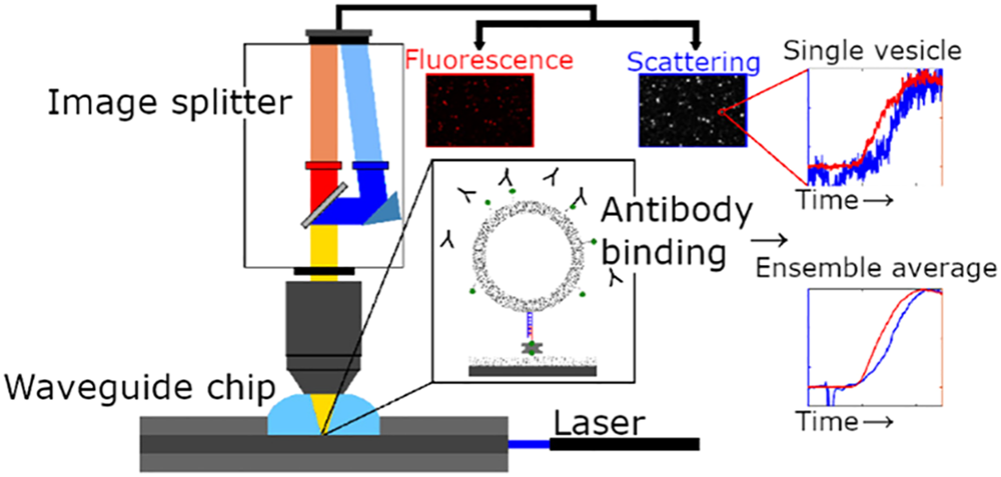

In-depth understanding of the intricate interactions between biomolecules and nanoparticles is hampered by a lack of analytical methods providing quantitative information about binding kinetics. Herein, we demonstrate how label-free evanescent light-scattering microscopy can be used to temporally resolve specific protein binding to individual surface-bound (∼100 nm) lipid vesicles. A theoretical model is proposed that translates protein-induced changes in light-scattering intensity into bound mass. Since the analysis is centered on individual lipid vesicles, the signal from nonspecific protein binding to the surrounding surface is completely avoided, offering a key advantage over conventional surface-based techniques. Further, by averaging the intensities from less than 2000 lipid vesicles, the sensitivity is shown to increase by orders of magnitude. Taken together, these features provide a new avenue in studies of protein-nanoparticle interaction, in general, and specifically in the context of nanoparticles in medical diagnostics and drug delivery.

Nanoparticles (NPs) of either synthetic or biological origin play key roles in a multitude of biological processes, such as viral infection and intercellular communication, as well as in many biotechnical applications, where exosomes, viruses, and synthetic NPs are used for drug or vaccine delivery and local treatment. Increasing attention has recently been paid to the formation of a so-called protein corona on NPs exposed to complex biological environments.^1^ This process may involve both specific biomolecular interactions with ligands present on the surface of NPs^2^ or unspecific interactions,^3^ which both can influence NP degradation, cellular uptake, and endosomal processing.^4^ The analytical methods currently available to study such interactions suffer from various limitations. Electron microscopy (EM) and atomic force microscopy (AFM) offer high quality structural information,^5,6^ while being limited with respect to statistics and the possibility to resolve interaction kinetics. Nanoparticle tracking analysis (NTA)^7^ and flow cytometry (FC)^8^ provide high-quality statistics but are restricted to NP size determination, in the case of NTA, and subpopulation identification using fluorescent markers in the case of FC, and neither approach is well suited for investigating kinetics. In contrast, high quality statistics and interaction kinetics can be efficiently obtained using surface-based fluorescent microscopy methods with single-NP resolution, such as total internal reflection fluorescence (TIRF) microscopy,^9^ but in analogy with FC, it requires labeling of the investigated molecules. Ensemble-averaging surface-based methods, like quartz crystal microbalance with dissipation (QCM-D) monitoring and surface plasmon resonance (SPR),^10^ including localized SPR,^11^ provide label-free readout and offer kinetic information but lack instead single NP resolution. Considering the fact that biological NPs often have broad distributions in terms of composition, structure, and size, and that the associated biomolecular interaction kinetics strongly depends on such properties and therefore may exhibit features that are hidden at the ensemble level,^12^ there is a need for methods that operate at the single-NP level to enable investigation of sample heterogeneity, while simultaneously providing label-free quantitative readout with sufficient statistics and temporal resolution to enable investigations of interaction kinetics.

Here, we take a step toward addressing the challenge of quantitative characterization of protein binding kinetics to surface-bound NPs using evanescent waveguide microscopy,^13^ enabling real-time signal acquisition from simultaneously recorded fluorescence and label-free light-scattering signals. The waveguide platform allows for substantial reduction of the background signal by matching the refractive index of the waveguide cladding to the protein-containing buffer solution, providing label-free observation of the scattering signal from individual surface-bound NPs.^14^

To demonstrate the application of this technique in the context under consideration, we measured specific binding of streptavidin (SA) and antibiotin-IgG antibodies (antibiotin) to surface-bound biotin-modified lipid vesicles. To verify that the observed changes in the scattering signal originate from protein binding, both proteins were fluorescently labeled, which in addition enabled a direct comparison between the signal-to-noise ratios for the two modes of operation. Complementary measurements for the same system were performed using dual-wavelength SPR,^10^ which verifies both the accuracy of the scattering microscopy measurements and the theoretical model developed to translate changes in the scattering signal upon protein binding into bound mass. Emphasis is also put on the possibility to investigate both sample heterogeneity and improvement of the signal-to-noise ratio by integrating the scattering intensity from multiple NPs. Combined with the possibility of using image analysis to exclude the background signal from nonspecific interactions with the surface surrounding the NPs, we demonstrate how label-free scattering microscopy could become an attractive alternative to conventional ensemble-averaged surface-based methods.

In our experiments, POPC vesicles containing 5 mol % biotin-modified lipids were tethered via cholesterol-modified DNA to the waveguide surface as described elsewhere^15^ (see inset in Figure 1 and Materials and Methods in the SI). Binding of individual vesicles was observed using live monitoring of their scattering intensities, allowing the tethering process to be terminated before crowding at the surface prevented individual particles to be distinguished (∼0.2 vesicles/μm^2^). Subsequently, the scattering and fluorescence intensities of individual vesicles were simultaneously recorded upon exposure to fluorescently labeled protein (SA or antibiotin) using an image splitter consisting of a dichroic mirror and optical filters arranged to decouple the scattered light from the emitted fluorescence signal (Figure 1).

**Figure 1:**
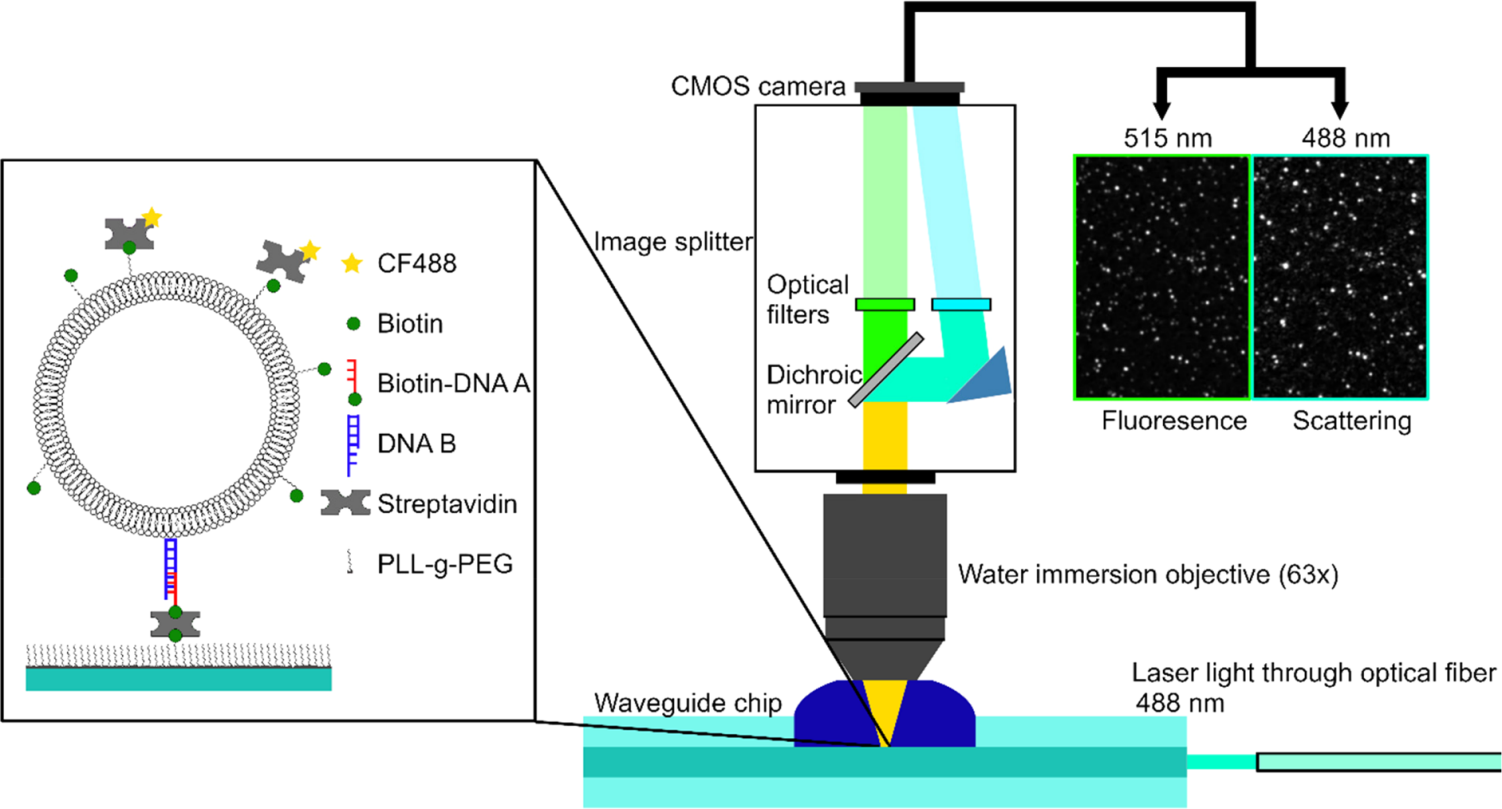
Scheme of the microscopy setup for simultaneous monitoring of the scattering and fluorescence signals of individual lipid vesicles. The POPC vesicles containing 5 mol % DSPE-PEG-biotin were linked to the waveguide surface using cholesterol-DNA-biotin tethers bound to SA on surface-immobilized PLL-*g*-PEG-biotin. The binding of either fluorescently labeled streptavidin CF488-SA or CF488-antibiotin molecules was then monitored in both scattering and fluorescence mode. The corresponding micrographs (approximately 20 μm wide) show the signals after protein binding.

Representative scattering and fluorescence signals from single vesicles upon exposure to CF488-SA (18 nM) or CF488-antibiotin (6 nM) are shown in Figures 2a and 2b, respectively. Both signals increase monotonically until saturation is reached, after which a slight decrease is seen in the fluorescence intensity. The data clearly display the differences in the intrinsic nature of the two signals, with the scattering signal displaying a factor of 2 to 3 larger high-frequency fluctuations than the fluorescence signal. These fluctuations are predominantly uncorrelated between the two signals. Thus, the fluctuations in the scattering mode do not primarily stem from alterations in the excitation intensity; both stochastic vesicle motion within the evanescent field and changes in vesicle shape are expected to have a higher impact on the scattering compared to the fluorescence. The observed reduction in fluorescence near saturated binding is attributed to a combination of photobleaching and self-quenching at high protein coverage.

**Figure 2:**
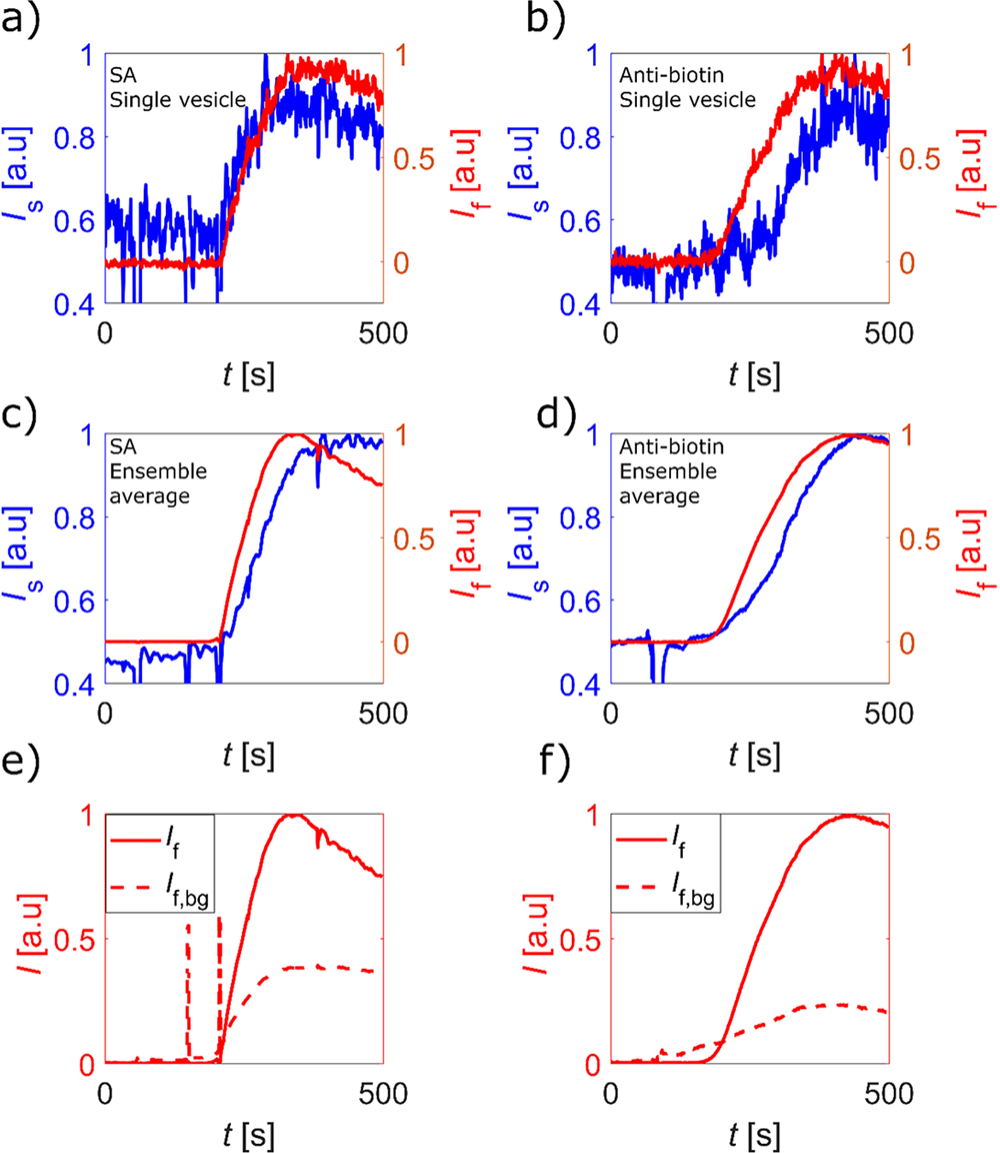
Normalized waveguide-microscopy intensities: scattering (blue) and fluorescence (red), as a function of time for vesicles modified with 5 and 3 mol % biotin-lipids upon exposure to (a) and (c) CF488-streptavidin (18 nM) and (b) and (d) CF488-antibiotin (6 nM), respectively. (a) and (b) show the signals for representative single vesicles, and (c) and (d) exhibit the ensemble-averaged signal using around 1700 vesicles in each experiment. Since (a) and (b) represent single vesicle data while (c) and (d) represent average values, the absolute signals differ slightly. The same fluorescence data as in (c) and (d) are shown in (e) and (f) also including the background signal from the area between the vesicles (*I*_f,bg_). The sudden spikes in intensity seen in (c), (d), and (e) are related to liquid injection and/or mixing.

In addition, the temporal changes in scattering and fluorescence intensity collected from individual vesicles can be integrated, resulting in an ensemble-averaged response (Figures 2c and 2d), in principle providing the same information as in ensemble-averaging surface-sensitive measurements. The integrated signals from around 1700 vesicles upon SA and antibiotin binding demonstrate a significant reduction in signal fluctuations compared to the single-vesicle traces, with high-frequency signal-to-noise of around 1800 and 110 in the fluorescence and scattering modes, respectively. Further, with local background subtraction and by restricting the analysis to the vesicles only, unspecific signals due to protein binding to the surface between the vesicles are efficiently filtered out in a way not possible using conventional ensemble-averaging surface-based methods. This is exemplified in Figures 2e and 2f, in which the ensemble-averaging fluorescence results shown in Figures 2c and 2d are compared with the unspecific response originating from fluorophores present in the area between the vesicles. Even though special care was taken to reduce unspecific adsorption using PLL-*g*-PEG surface functionalization,^16^ the unspecific signal is seen to be appreciable (10–20%). To rule out the possible influence of fluorescence on the scattering signal, additional measurements were conducted using unlabeled SA, which showed nearly identical results as obtained using the labeled SA (Figure S1 in the Supporting Information). Although the high-frequency fluctuations are smaller for the fluorescent signal, the decrease in the fluorescence signal due to photobleaching and/or self-quenching is substantial, highlighting a key limitation of fluorescent labeling.

The theoretical basis for the interpretation of measurements performed in fluorescence and scattering modes has already been developed (see, e.g., refs (9), (12, and 13), respectively). For the measurements of protein adsorption to lipid vesicles, a slight extension is, however, needed. The scattering intensity of a surface-bound NP subjected to evanescent light can be represented as
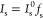
1where
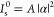
2is the intensity calculated in the Rayleigh limit neglecting light extinction and phase shifts (α is the NP polarizability, and *A* is a function that includes the square of the evanescent field intensity), and *f*_s_ is a dimensionless correction factor taking extinction and phase shifts into account. In our model, a lipid vesicle is viewed as a spherical shell of outer radius *r* and lipid-shell-thickness *d*, the effective thickness of which is increased by Δ*d* upon protein binding. Without protein, the polarizability for a vesicle is (see eq 5.36 in ref (17) at *d* ≪ *r*)
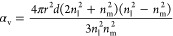
3where *n*_l_ and *n*_m_ are the lipid and medium refractive indices, respectively (the refractive index of the medium is assumed to be equal to that of the vesicle interior). Upon formation of a protein layer of thickness Δ*d* ≪ *r*, the polarizability of the vesicle-protein system is
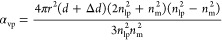
4where *n*_lp_^2^ = (*n*_l_^2^*d* + *n*_p_^2^Δ*d*)/(*d* + Δ*d*) is the effective refractive index of the shell (this is the simplest reasonable expression for the effective refractive index of a mixture,^18^ where *n*_p_ is the protein refractive index). To determine Δ*d*, we can use the ratio of the increment of the scattering intensity upon protein attachment, Δ*I*_s_ ≡ *I*_s,vp_ – *I*_s,v_, and the scattering intensity of a vesicle without protein, *I*_s,v_, where *I*_s,vp_ represents the scattering intensity after protein binding. For thin spherical shells, the correction factor *f*_s_ depends on *r* only. Hence, in our case, *f*_s_ can to a first approximation be assumed to be equal for the situations with and without protein, which in turn means that we have
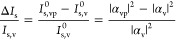
5Applying this relation in combination with eqs 3 and 4 to our experimental observations (Figure 2), using *d* = 4.5 nm for the lipid membrane thickness^12^ and *n*_m_ = 1.335,^19^*n*_l_ = 1.49, and *n*_p_ = 1.60 (with the refractive index values calculated^20^ from the respective refractive index increment values of lipids^21^ and proteins,^22^ respectively) yields Δ*d* = 1.57 ± 0.16 nm and Δ*d* = 1.74 ± 0.6 nm for SA and antibiotin, respectively.

Further, the protein layer thickness Δ*d* can be related to surface mass concentration Γ through de Feijter’s formula^23^
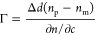
6where *∂n*/*∂c* is the derivative of the refractive index with respect to protein mass density. Using this relation in combination with *∂n*/*∂c* = 0.185 mL/g (ref (21)) and the *Δd* values obtained above, we have for SA and antibiotin Γ values of 225 ± 23 and 249 ± 86 ng per cm^2^ membrane area, respectively. With molecular weights of ∼56 and 150 kDa and a vesicle radius of 52.5 nm, this converts to 838 ± 86 and 346 ± 119 protein molecules per vesicle, respectively. With footprint areas of ∼20 nm^2^ for SA and 35 nm^2^ for antibiotin, this corresponds to surface coverages of 48% and 35%, respectively, which are close to the jamming limit of ∼54%. These numbers make it possible to quantify the detection limit in terms of number of proteins per vesicle, which for single vesicle data (Figures 2a and b) becomes around 120 and 40 for SA and IgG, respectively, and a factor of around 15 lower after ensemble averaging.

The effective thickness, as defined above, corresponds to a dense protein film with *n*_p_ = 1.6. In reality, the film thickness is expected to match the molecular dimension of the proteins (around 5 and 15 nm for SA and antibiotin, respectively), with a corresponding reduction in the refractive index of the film. Using these thickness values, eqs 2–5 yield effective refractive index values, *n*_eff_, of 1.44 and 1.38 for SA and antibiotin. Although this is significantly lower than the *n*_p_ value of ∼1.6 obtained from the *∂n*/*∂c* of proteins, an increase in film thickness keeps the mass fairly conserved when estimated using eq 6 and corresponds to 221 and 245 ng per cm^2^ membrane area for SA and antibiotin, respectively (see the Supporting Information for further details).

While the quantitative analysis above was made for the ensemble-averaged data, it is also instructive to perform the corresponding analysis on a single-vesicle level, since this clarifies distributions and subpopulations of the samples. Since the scattering intensity scales with mass volume squared (provided *f*_s_ is not too small), *I*_s,v_ ∝ *r*^4^*d*^2^ for a spherical vesicle with *d* ≪ *r* (see eqs 2 and 3), and under the assumption that *d* is independent of *r*, *I*_s,v_^1/4^ reflects the distribution in vesicle radius. Plotting the surface concentration, Γ, as a function of *I*_s,v_^1/4^ (Figure 3) thus offers a way to visualize the distribution of mass as a function of vesicle size. Even though the distribution in Γ is fairly broad for both SA and antibiotin, reaching from around 100 to 700 ng per cm^2^ lipid membrane area, with a mean surface concentration around 200 ng/cm^2^ for both proteins, the data seem to indicate a slight tendency toward higher protein coverage for smaller vesicles for both types of proteins (Figures 3a and b). Despite this apparent difference in protein coverage, the binding kinetics is essentially identical for small and large vesicles for both proteins, as illustrated in Figures 3c and 3d for SA and antibiotin, respectively. Under the reasonable assumption that the biotin coverage is the same on small and large vesicles, a significant difference in bound mass is expected to be reflected in different kinetic profiles. The similar kinetic traces thus suggest that the quantification of bound mass is not reliable for the faintest vesicles, leading to an overestimation of Δ*I*_s_/*I*_s,v_ for small *I*_s,v_. This interpretation is further supported by the fact that the fluorescence signal increases with *I*_s,v_, (see Figure S3). By comparing the distribution in *I*_s,v_^1/4^ with the radii obtained from nanoparticle tracking analysis of the same vesicle batches (Figures 3a and 3b), one can also conclude that the overestimation Δ*I*_s_/*I*_s,v_ becomes appreciable at vesicle diameters below ∼70 nm, while the actual detection limit is ∼25 nm.

**Figure 3:**
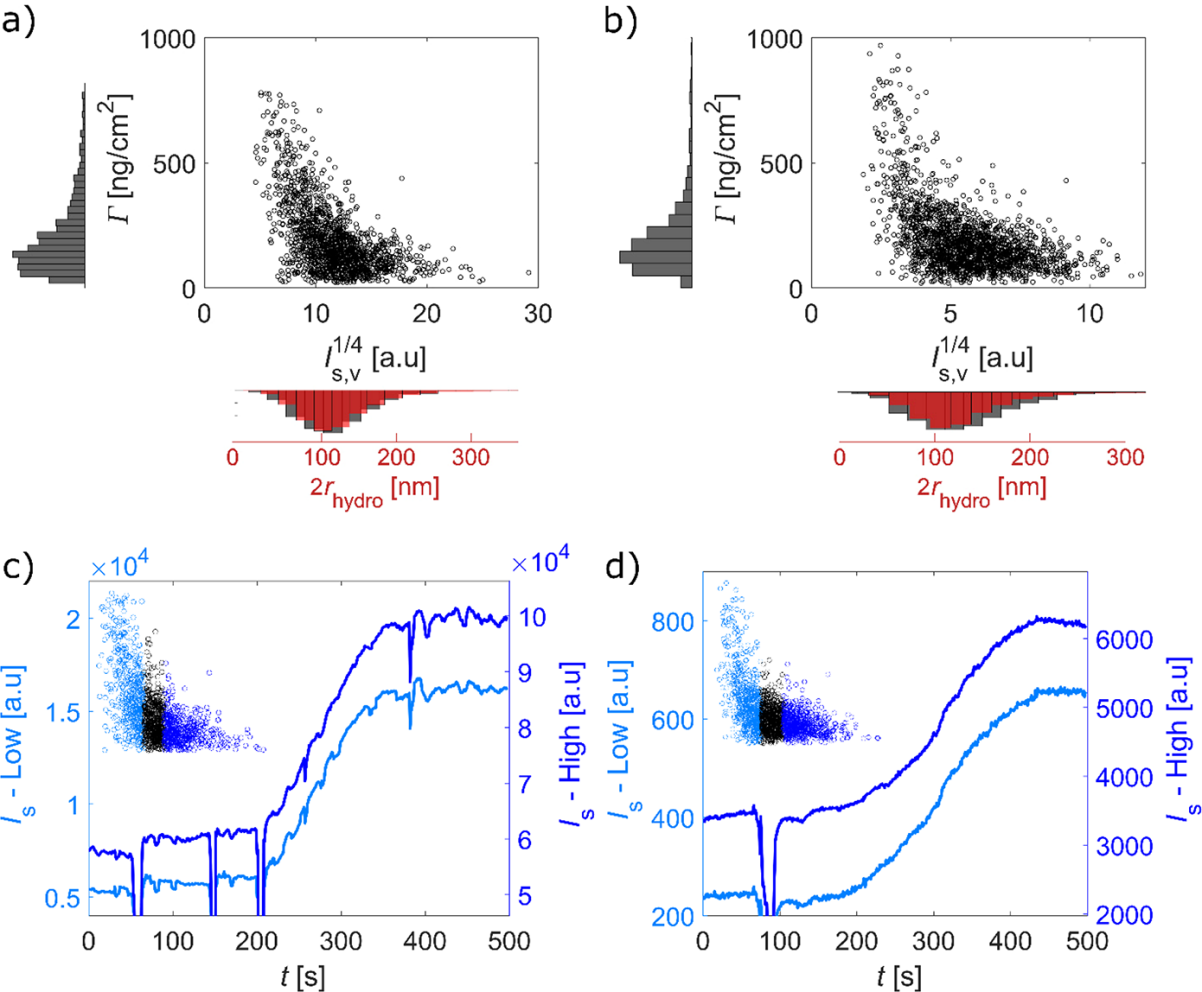
Calculated adsorbed protein surface concentration, Γ, expressed in terms of mass per cm^2^ of lipid membrane, as a function of the fourth root of the initial vesicle scattering intensity, which is proportional to the vesicle diameter, for (a) SA and (b) antibiotin binding to biotinylated vesicles, with the corresponding distributions projected onto the axes, including the size distribution determined in bulk using NTA plotted together with the distribution in *I*_s,v_^1/4^. (c) and (d) show, for binding of SA and antibiotin, respectively, the ensemble-averaged scattering intensity versus time of the fraction (33%) of the vesicles with the lowest (*I*_s_ -Low, light blue) and highest (*I*_s_ -High, dark blue) initial scattering intensity. The sudden reductions in intensity are related to liquid injections and/or mixing. The scattering intensity represents the measured values after background subtraction (see Section 1 in the Supporting Information) which varies depending on illumination intensity and in-coupling efficiency.

To compare the absolute mass quantification based on light scattering with a method well established for mass-uptake quantification, we used dual wavelength SPR.^10^ The corresponding SPR response upon SA binding to tethered lipid vesicles of the type used above (Supporting Information, Section 1 and Figure S2a,b) yields a protein mass coverage of ∼220 ng per cm^2^ membrane area or ∼850 streptavidin molecules per vesicle (Table 1). The corresponding SPR data for antibiotin binding (Figure S2c,d) convert to a surface-mass concentration of 380 ng per cm^2^ membrane area or 470 antibiotin molecules per vesicles (Table 1). These values are in good agreement with the scattering data, and the small deviations are likely due to somewhat different experimental conditions for the two systems (liquid handling, different biomolecule concentrations).

**Table 1:** Summary of the Scattering Microscopy and SPR Data Including the Mass of Adsorbed Vesicles, the Measured Signal upon SA and Antibiotin Binding Normalized to the Signal for the Immobilized Vesicles, and the Number of Proteins Per Vesicle Obtained for the Respective Method

	SA/vesicle (relative signal)	SA/vesicle (#)	IgG/vesicle (relative signal)	IgG/vesicle (#)
scattering microscopy	1.26	838	1.59	346
SPR	0.64	850	1.06	470

In conclusion, using label-free evanescent light-scattering microscopy to measure the kinetics of protein binding to individual surface-tethered lipid vesicles and by applying an analytical model, we have successfully quantified the mass uptake of streptavidin and antibiotin. The temporal evolution of the scattering and fluorescence signals is observed to differ significantly (Figure 2), which is attributed to the fact the fluorescence signal scales with the number of bound proteins, while the scattering signal scales as the square of the volume (mass) of the scattering objects (see eqs 2 and 3). This was confirmed by converting the temporal evolution of the scattering signal into bound mass, Γ, providing essentially linear curves when plotted versus the change in the fluorescence signal, *I*_f_, for SA (Figure S4a), thus verifying that binding kinetics can be reliably extracted from the scattering signal. In the case of antibiotin, the correlation between Γ and *I*_f_ displays a slight deviation from linearity (Figure S4b), which can be attributed to the larger dimension of the IgG antibodies which may act to induce changes in the structure of the protein film as the coverage increases,^12^ thus affecting the scattering signal. Effects of fluorescence quenching may also play a role, resulting in the fluorescence intensity not scaling linearly with the number of bound proteins at higher coverages.

The protein mass quantification was verified by complementary SPR measurements, which represents a well-established means to quantify adsorbed protein mass. Compared to SPR, the waveguide data are based on single vesicle measurements, revealing a distribution in both vesicle size that provides information about potential heterogeneities in both binding kinetics and size-dependent binding efficiency (Figure 3). However, by integrating the scattering response from all measured vesicles, the single-vesicle data can be transformed into an ensemble-averaged response, which is, in principle, analogous to that obtained using SPR, with a few crucial differences.

First, since the signal response in standard ensemble-averaged methods originates from any type of surface interaction, the use of surface passivation or high surface coverage is required to decrease effects of unspecific binding to surface regions surrounding the NPs. In contrast, the possibility in scattering microscopy to resolve each individual vesicle makes it possible to completely omit the surface exposed between the vesicles from the analysis, thus reducing the effect of unspecific binding to virtually zero (Figure 2e,f). Identification of efficient antifouling surface treatments is even more challenging in many practical situations and, in particular, when biosensing is carried out in complex biological media such as serum.^24^ This makes methods that allow for complete elimination of nonspecific binding an important complement to the assortment of surface analytical tools.

Second, attaining high surface coverage of the investigated entities required for methods like SPR is not always possible, especially not when studying protein binding to native biological NPs, such as extracellular vesicles or viruses, which are often available in low amounts in biological fluids. The single-NP sensitivity of scattering microscopy allows for approximately 3 orders of magnitude lower vesicle surface coverage to be used than required for the SPR measurements but with a comparable signal-to-noise ratio when averaging over all vesicles in the field of view.

It is also worth comparing the limit of detection obtained for the single-NP resolved waveguide scattering and fluorescence microscopy, which corresponds to approximately 30 and 10 antibiotin proteins, respectively. These results are encouraging from the perspective of utilizing spatiotemporally resolved scattering microscopy of NPs as a label-free tool for studying biomolecular binding kinetics. It is worth noting, though, that in the present setup, protein was added to a liquid droplet placed on top of the waveguide chip, followed by rapid mixing. An estimate of the time scale characterizing relaxation of the solution motion after mixing (<1 s) and the protein flux during binding suggest that the measured kinetics is limited by global diffusion (see Section 5 in the Supporting Information). Inspection of reaction controlled binding kinetics with single nanoparticle resolution would benefit from designs that integrate microfluidics to enable liquid exchange similar to that used in, for example, SPR. Use of transparent rather than opaque silicon chips would also facilitate microfluidic handling and make it possible to investigate turbid samples by employing inverted microscopes.

Taken together, the results presented in this work demonstrate that scattering microscopy with single-NP resolution has the potential to complement the existing arsenal of label-free surface-sensitive methods, of particular relevance in the context of protein interaction with NPs of biological origin, which are often available at low concentration and frequently analyzed in complex biological fluids, in which case nonspecific surface binding tends to further complicate the analysis. Hence, with the rapidly growing interest in biological NPs for drug and vaccine delivery, cancer treatment, as well as nanosafety, we foresee a wide utility for this measurement approach.

## Supporting Information Available

Data from scattering experiments using unlabeled protein, dual-wavelength SPR experiments, and comparison between changes in scattering and fluorescence signal upon protein binding (PDF)
