# Supplementary material for: Time-Resolved and Label-Free Evanescent Light-Scattering Microscopy for Mass Quantification of Protein Binding to Single Lipid Vesicles
Source: Nano Lett. 2021 May 18;21(11):4622–8. doi: 10.1021/acs.nanolett.1c00644 (PMC8289281; doi:10.1021/acs.nanolett.1c00644)
Supplement: Supplementary file 1 — nl1c00644_si_001.pdf [file nl1c00644_si_001.pdf]

Supporting information

Time-Resolved and Label-Free Evanescent-Light-Scattering Microscopy for Mass Quantification of Protein-Binding to Single Lipid Vesicles

*Mattias Sjöberg[a], Mokhtar Mapar[a], Antonius Armanious[a] i, Vladimir P. Zhdanov[a,b], Björn Agnarsson[a], and Fredrik Höök[a]**

[a] Division of Nano and Biophysics, Department of Physics, Chalmers University of Technology, Gothenburg, Sweden

[b] Boreskov Institute of Catalysis, Russian Academy of Sciences, Novosibirsk, Russia

i Present address: Laboratory of Food and Soft Materials, Department of Health Sciences and Technology, ETH Zürich, Zürich, Switzerland

*Corresponding author: fredrik.hook@chalmers.se

1. Materials and Methods

Preparation of vesicles

Biotinylated vesicles were made of 95 mol% 1-palmitoyl-2-oleoyl-glycero-3-phosphocholine (POPC) and 5 mol% 1,2-distearoyl-sn-glycero-3-phosphoethanolamine-N- [biotinyl(polyethylene glycol)-2000] (DSPE-PEG(2000)-biotin) lipids purchased from Avanti Polar lipids. For the anti-biotin binding, a POPC:DSPE-PEG(2000)-biotin molar ratio of 97:3 was used. Lipid vesicles were produced by forming a lipid film in a round-bottom flask from lipids dissolved in chloroform by drying in a rotary evaporator and subsequent further vacuum-drying for 8 hours. The film was rehydrated through the addition of phosphate buffered saline (PBS) solution (150 mM, pH 7.4, Sigma-Aldrich) in quantities yielding a 1 mg/ml lipid concentration. The vesicles were subjected to five cycles of freeze-thawing using liquid nitrogen and a 40°C water bath and subsequent extrusion through a polycarbonate membrane (100 nm pores, Whatman) 31 times.

Labelled streptavidin and anti-biotin

Labelled and unlabeled streptavidin (55 kDa, $5.6 \times 5 \times 4$ nm^3[1]), which is commonly used for its high affinity and selectivity to biotin, was purchased from Sigma-Aldrich, aliquoted in PBS and stored at -25 °C. Monoclonal antibiotin-IgG antibodies (150 kDa) were purchased from Sigma-Aldrich, aliquoted in PBS and stored at -25 °C.

DNA-tethers

The DNA tethers (Eurogentec), used for surface binding of vesicles, consisted of three parts, which when incubated together for 30 min, produce a 30 base pair long double stranded tethers consisting of two cholesterol moieties in one end and biotin in the other. The parts used had the following sequences:

5’-TGG-ACA-TCA-GAA-ATA-AGG-CAC-GAC-GGA-CCC-3’-TEG-Cholesterol

Cholesterol-TEG-5’-CCC-TCC-GTC-GTG-CCT-3’

5’-TAT-TTC-TGA-TGT-CCA-CCC-CC-3’-TEG-Biotin

Waveguide microscopy measurements

The manufacturing of the waveguide chips is described in detail elsewhere [2]. Prior to use, the surface of the waveguide chip was rinsed in ultrapure H_2O, dried in N_2 and O_2 plasma treated (30 W, Harrick Plasma cleaner) for 5 minutes. The chip was then sequentially incubated for 30 min with: i) 50 µg/mL Poly(l-lysine)-graft-poly(ethylene glycol) (PLL(20)-g [3.5]- PEG(2), SuSoS

AG), of which 5% was functionalized with biotin, ii) 40 µg/mL of streptavidin and iii) 39 nM biotinylated DNA-tethers. The chip was rinsed with PBS between each step to wash away any excess material. The chip was subsequently placed under an upright Olympus BX61 microscope equipped with a 60X, NA 1.0 water-dipping objective, a Hamamatsu ORCA-Flash 4.0 V2.0 CMOS camera, and a Hamamatsu W-VIEW GEMINI image splitter, containing specific filter cubes (dichroic mirror: 510 nm, fluorescence bandpass: 535/50 and scattering bandpass: 488/10) allowing for simultaneous acquisition of fluorescence and scattering signals. Illumination was achieved using a 488 nm, Cobolt, 06-01 series, CW, diode laser source, coupling TE-polarized light into the waveguide core via a single-mode polarization maintaining optical fiber. The measurements were conducted with the laser set to 6 mW and data recorded at 2 frames per second, with camera exposure set to 100 ms. A vesicle solution of low concentration was added to the chip, resulting in a thickness of the solution of approximately 3 mm between the chip and the objective. Vesicle tethering to the surface was observed in real-time until a surface coverage of approximately 1000 vesicles per field of view was achieved, after which the incubation step was interrupted by rinsing. After buffer rinsing, a solution containing the respective protein was added to the buffer, with a final protein concentration of $\approx$1 µg/mL for either the CF488-labeled streptavidin (Biotium) or the CF488-labelled anti-biotin. The total number of bound proteins, based on the measured vesicle and protein surface mass concentrations, amounts to approximately 0.1% of the total protein content in solution.

Data Extraction from Waveguide Microscopy Measurements

Measurements conducted as described above, in which a solution of fluorescently labeled proteins was added to surface immobilized vesicles, produced a stack of images which were processed as follows: The images representing scattering data were registered over time through a Fourier transform-based phase correlation method [3], after which the fluorescence images were aligned to their scattering counterparts, taking translation, rotation, scale and shear-transformations into account. The vesicles were identified and distinguished from background by locating local maxima in a fluorescence image after protein binding. To reduce effects of noise and background, the images were subsequently processed by Gaussian filtering and morphological image opening. To estimate the scattering and fluorescence intensity attributed to individual vesicles, an area covering each identified particle and an associated local background area free of particles were selected, after which the pixel intensity values minus the average local background

was integrated for every particle. To produce Figure 2 in main text, time resolved single-vesicle and ensemble-average intensity plots were normalized to their respective maximum intensity values, while the background intensity plots were normalized to their respective non-background maxima. To calculate the bound protein mass based on the recorded intensity values, the temporally resolved scattering intensity data before protein addition and after saturated protein binding were noise-reduced using Gaussian and rolling window median smoothing (~50 to 200 data points), after which $I_{\mathrm{s,v}}^{0}$ and $I_{\mathrm{s,vp}}^{0}$ were determined from the average or maximum intensity values, respectively. The bound mass attributed to each individual vesicle was then calculated using to the equations described in the main text and plotted versus the fourth root of the initial scattering intensity (Figure 3 in main text).

SPR measurements

The dual-wavelength SPR measurements were performed using an SPR Navi 220A instrument (BioNavis), utilizing wavelengths 670 nm and 785 nm, a scanning interval of 58-78 degrees and SiO_2-coated sensors (SPR102-SIO2, BioNavis). The sensors were prepared by sonication in a bath sonicator for 15 min (ultrasonic frequency, 45 kHz; ultrasonic power level, 60%, VWR USC-THD) in 2%wt. sodium dodecyl sulfate (SDS, Sigma-Aldrich), rinsing with ultrapure H_2O (Synergy systems, Merck Millipore Corporation), drying under N_2 flow followed by O_2 plasma treatment (Harrick Plasma cleaner, 30 W) for 3 minutes. The measurements were run at 20 µL/min at 25 °C using PBS (150 mM, pH 7.4, Sigma-Aldrich) as buffer. After establishing a baseline under the flow a PBS buffer, the sensor surface was sequentially exposed to the following 30 min functionalization steps: 50 µg/mL Poly(l-lysine)-graft-poly(ethylene glycol) (PLL(20)-g [3.5]-PEG(2), SuSoS AG), of which 5% was functionalized with biotin; 40 µg/mL of streptavidin, 39 nM biotinylated DNA-tethers; 100 µg/mL of vesicle solution and 20 µg/mL of CF488-labeled streptavidin (Biotium). Each of these steps was followed by rinsing with PBS. The system sensitivity was gauged by exchanging the regular H_2O based PBS with a buffer identically prepared in D_2O. The system decay lengths were determined in separate experiments where a DOPC supported lipid bilayer was formed on the sensor surface. Assuming a bilayer thickness of 4.5 nm, corrections to the theoretically calculated decay length values δ_{λ} (see later section) were determined from R_{670}/R_{785}.

Nanoparticle Tracking Analysis

The vesicle size distribution was measured using a NanoSight LM10 NTA module (Malvern Instruments Ltd., United Kingdom; 488 nm laser) at room temperature under steady flow conditions using a NanoSight syringe pump.

2. Scattering data for non-labeled streptavidin

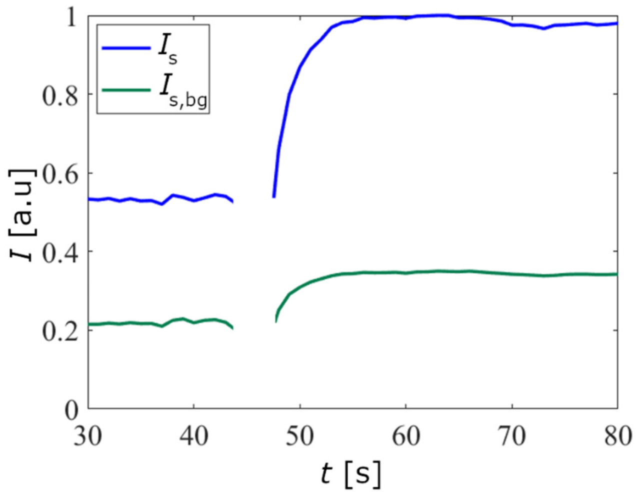

Figure S1: Temporal evolution of the normalized waveguide-microscopy scattering intensity I_s (ensemble average signal using around 600 vesicles) and the background scattering intensity $I_{s,bg}$ (ensemble average signal from the area between the vesicles) for vesicles modified with 5 mole% biotin-lipids upon exposure to non-labeled streptavidin (18 nM). The fall in the signal during liquid injection is omitted (t ~45-47 s).

3. Dual wavelength SPR

Absolute mass quantification of protein binding to lipid vesicles immobilized using the same protocol as that used for the waveguide-obtained data presented in the main text was made using dual wavelength SPR [4]. The SPR response, R_λ, was monitored at two wavelengths in real time upon tethering of biotinylated vesicles (1.33 nM) to a streptavidin functionalized sensor surface followed by subsequent rinsing and exposure to either CF488-SA (360 nM) or anti-biotin-IgG (133 nM). The protein coverage per vesicle was then deduced from the response signal R_λ via standard means.

Dual wavelength SPR also allows the thickness, l, of the lipid vesicle layer or, in other words, the vesicle diameter (provided vesicles are spherical) to be quantified without or with protein by presenting the ratio of the SPR responses measured at the two wavelengths [4],

$$\frac{R_{\lambda_1}}{R_{\lambda_2}} = \frac{S_{\lambda_1}\, \partial n/\, \partial c_{\lambda_1}\left(1 - \exp\left[-l/\delta_{\lambda_1}\right]\right)}{S_{\lambda_2}\, \partial n/\, \partial c_{\lambda_2}\left(1 - \exp\left[-l/\delta_{\lambda_2}\right]\right)} \quad \text{(S1)}$$

where S_{λ_i} are sensitivity factors and δ_{λ_i} the decay lengths of the respective evanescent field intensities. S_{λ_i} were obtained by alternating between H_2O and D_2O based buffer solutions and measuring the responses while δ_{λ_i} and $\partial n/\partial c_{\lambda_i}$ were derived theoretically [5] and from tabulated values [6], respectively. The values are summarized in Table S1.

Table S1 Sensitivity factors, penetration depths and refractive index increments for different wavelengths for the BioNavis the SPR instrument. Sensitivity factors were obtained by measuring responses in two different liquids of known refractive indices; the penetration depths were estimated theoretically [5] and the refractive index increments were obtained from tabulated values [3].

λ_i [nm]	S_{λ_i}(SA) [RIU/nm]	S_{λ_i}(IgG) [RIU/nm]	δ_{λ_i}[nm]	$\partial n/\partial c_{\lambda_i}$[ml/g]
670	72	110	106	0.160
785	61	-	154	$1.02 \times \partial n/\partial c_{\lambda_{670}}$
980	-	82	267	$1.04 \times \partial n/\partial c_{\lambda_{670}}$

The temporal evolution of $R_{\lambda_i}/R_{\lambda_i}$ are indicated with black lines in Figures S2a and S2c for SA and anti-biotin-IgG binding, respectively, which via Eq. S1 can be converted to film thicknesses (Figures S2b and S2d). The measured response ratios upon vesicle binding ($R_{670}/R_{785} = 1.535$ and $R_{670}/R_{980} = 2.73$) indicate a mean vesicle diameter of a 100 ± 5 nm, which is in good agreement with the mean vesicle size diameter of 105 nm obtained through NP tracking analysis (see insets Figures S2b and S2d). It is also worth noting that although SA binding did not yield a detectable change in vesicle thickness through R_{670}/R_{785} (Figure S2a), the data clearly indicates negligible vesicle deformation upon protein binding, which confirms the validity of the scattering model outlined in Eqs. 1 to 6 in the main text.

The SPR data can be used to quantify both the vesicle coverage and the increase in mass by protein binding. Using a $\partial n/\partial c$ of 0.148 cm^3/g for vesicles and 0.16 cm^3/g as a mean value for

vesicles with bound proteins, the measured vesicle surface mass concentration corresponds to ~403 ng/cm^2 and the additional mass uptake upon SA binding is thus ~198 ng/cm^2. However, these numbers refer to the coverage on the planar SPR sensor surface, and hence, to make a comparison with the protein coverage per vesicle, as obtained from the scattering data, one needs to normalize to the mass of a planar bilayer. Assuming a bilayer thickness of 4.5 nm and a specific density of 1.004 g/ml [7], one gets 452 ng/cm^2 as the mass for a planar bilayer, which corresponds to a protein mass concentration of ~220 ng per cm^2 membrane area, or ~850 streptavidin molecules per vesicle (Table 1 in main text). The corresponding SPR data for anti-biotin-IgG binding to the same type of vesicles (Figures S2c and S2d) correspond to a surface mass concentration of 380 ng per cm^2 membrane area, or 470 anti-biotin-IgG molecules per vesicles. In the latter case, an increase in vesicle diameter obtained from Eq. S1 was also accounted for (see Figure S2d).

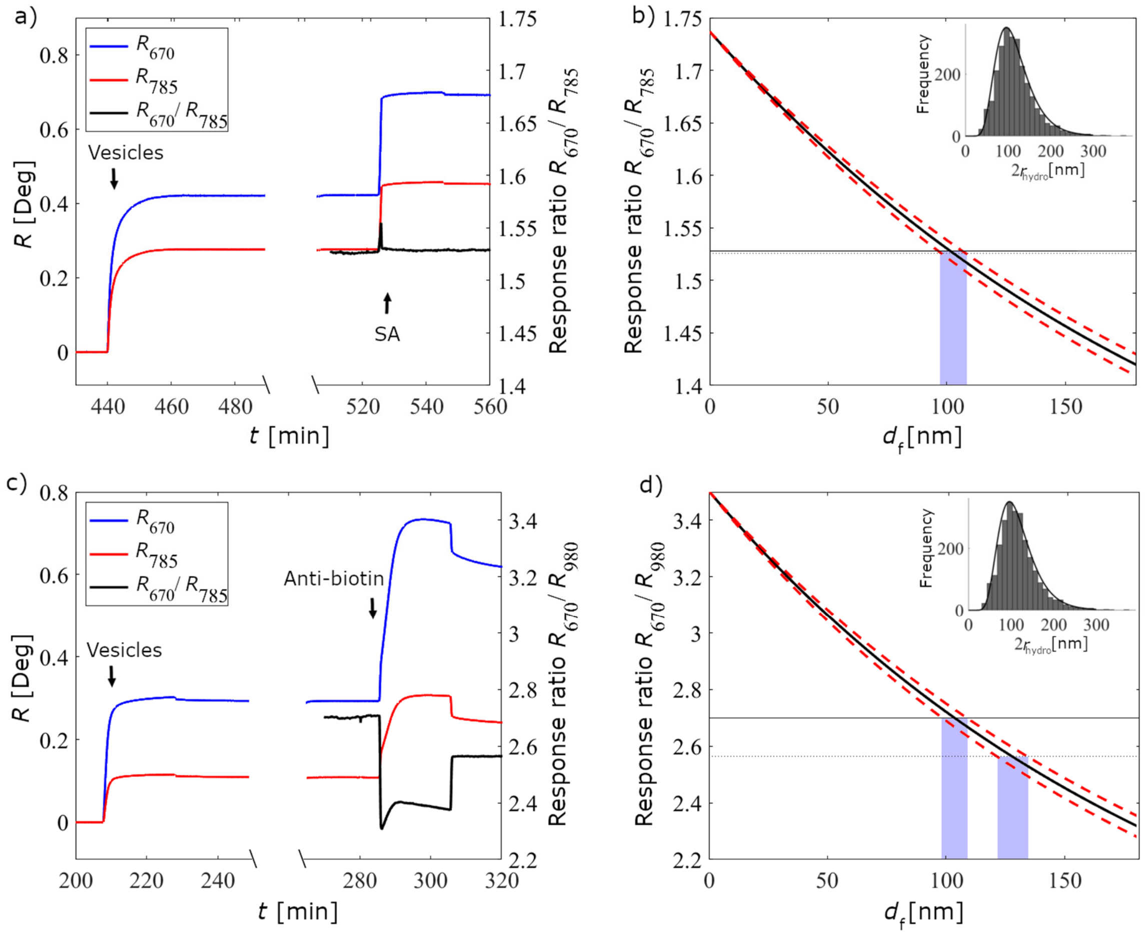

Figure S2: (a) The SPR response over time for the sequential addition of biotinylated vesicles and CF488-streptavidin (SA) to the flow cell. As the two wavelengths, 670 nm (blue) and 785 nm

(red), probe refractive index shifts at different sensing depths a considerable contrast emerges. The black line represents the ratio of the two responses. (b) The ratio R_{670}/R_{785} of the SPR responses as a function of adsorbed film thickness as expressed in Eq. S1. The measured response upon surface immobilization of biotinylated vesicles remained close to constant at 1.53 upon exposure to SA. The projection of the intercept with the model in Eq. S1 is indicated as a blue shaded area. The red dotted lines indicate the effect of a $\pm 5\%$ uncertainty in the determination of the respective decay lengths δ_λ. Inset: Histogram of vesicle diameter distribution as determined through nanoparticle tracking analysis (NTA). (c) and (d) show the corresponding data for anti-biotin-IgG binding measured with the SPR wavelengths 670 nm and 980 nm. The full and dotted horizontal lines in (d) correspond to the response ratio prior to and after protein injection. The corresponding two lines in (b) overlap.

Correction of the decay lengths for dual wavelength SPR

For thin films $(l \ll \delta)$, equation S1 can be approximated as:

$$\frac{\Delta R_{\lambda_1}}{\Delta R_{\lambda_2}} = \frac{S_{\lambda_1}(\partial n/\partial c)_{\lambda_1}\delta_{\lambda_2}}{S_{\lambda_2}(\partial n/\partial c)_{\lambda_2}\delta_{\lambda_1}} \quad \text{(S2)}$$

Since S_λ and $(dn/dc)_\lambda$ are known, the theoretical determinations of δ_{λ_2} and δ_{λ_1} can be compared with the $\frac{\delta_{\lambda_2}}{\delta_{\lambda_1}}$ ratio obtained from the SPR response upon formation of films significantly thinner than the decay length. For pure gold sensors it was previously shown[4] that the ratio is in good agreement with theoretically determined decay lengths of $\delta_{670} = 106$ and $\delta_{785} = 154$ nm; the sensors in the current work were however coated with a 10-20 nm silica film, a change expected to slightly influence the absolute values of the decay lengths. Since SLB formation fulfill the thin film approximation (l ~5 nm and the corresponding ratio, i.e. 1.75, matches with the output from equation (S1)), each sensor was calibrated based on the ΔR_{λ_1} and ΔR_{λ_2} responses upon SLB formation resulting in an estimated reduction in decay lengths of 6 to 11 nm for different sensors.

4. Comparison of the scattering and fluorescence signal

Although quantitative interpretation of fluorescence intensity is in general a fairly complicated task that tends to require careful calibration measurements and/or extensive knowledge and consideration of experimental setup-parameters and fluorophore characteristics [8], the fluorescence intensity, I_f, is expected to scale linearly with the number of bound proteins. First we

note that I_{f} increases with $I_{\mathrm{s,v}}$, (Figure S3). Although both quenching and bleaching make quantitative interpretation of these plots complicated on the level of individual vesicles, these graphs indicate that there is a correlation between the amount of bound protein and vesicle size ($I_{\mathrm{s,v}}{\sim}r^4$). This further supports that the seemingly higher protein coverage on small vesicles deduced from the scattering signal originates from overestimation of $\Delta I_{\mathrm{s}}/I_{\mathrm{s,v}}$ for small $I_{\mathrm{s,v}}$, although a higher protein coverage on small vesicles cannot be fully excluded.

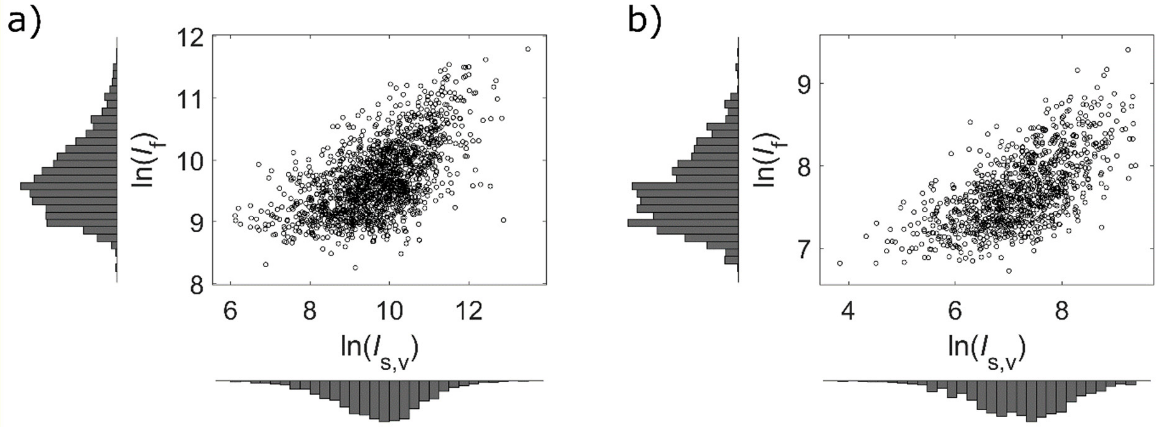

Figure S3: Logarithmic plot of the fluorescence intensity upon binding of labeled protein, I_f, as a function of vesicle scattering intensity prior to protein binding, $I_{s,v}$, for a) SA and b) anti-biotin, with the corresponding distributions projected onto the axes.

It is also worthwhile to note that I_{f} is expected to scale linearly with the protein mass per membrane surface area, Γ, calculated from the scattering intensity increase $\Delta I_{\mathrm{s}}/I_{\mathrm{s,v}}$ as described in the Results and Discussion section of the main text, while a deviation from linearity is expected between $\Delta I_{\mathrm{s}}/I_{\mathrm{s,v}}$ and I_{f}. This is illustrated in Figure S4, which displays $\Delta I_{\mathrm{s}}/I_{\mathrm{s,v}}$ and Γ plotted as a function of I_{f} for both SA and anti-biotin binding after correcting I_{f} for photobleaching and/or quenching effects. In the plots, Γ is determined using either a fixed protein refractive index n_p of 1.6 or a fixed thickness corresponding to the protein dimensions (5 and 15 nm for SA and anti-biotin, respectively). As expected, clear deviations from linearity are observed for $\Delta I_{\mathrm{s}}/I_{\mathrm{s,v}}$ versus I_{f}, while a more linear relation is observed for Γ for both film thicknesses. Note, however, that deviation from linearity for anti-biotin is greater than for the SA. Such deviations are partly related to the non-linear dependence of the scattering intensity on the particle mass and partly to other factors. For example, it may signal a protein coverage dependent variation in the homogeneity of

the protein distribution and thus symmetry of the scattering object, which was previously observed for α-synuclein induced vesicle deformation and rupture [7]. It might also be attributed to fluorescence quenching which is expected to increase with increasing surface coverage. In addition, the vesicle-size size distribution can here play a role, because the relative contributions of small and large vesicles to the signals change with increasing time and I_s and I_f for these vesicles are different. It should also be noted that although the actual protein mass concentration cannot be determined from the fluorescence signal alone, the quantitative nature of the scattering serves can serve to calibrate the fluorescence signal in terms of number of bound proteins.

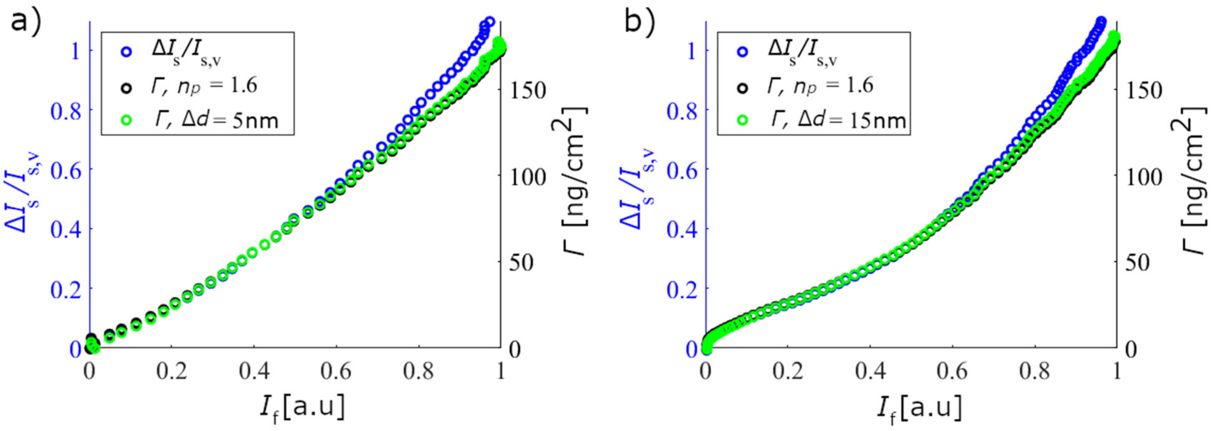

Figure S4: Scattering intensity increase, $\Delta I_s/I_{s,v}$ (blue), and bound protein mass per lipid membrane area, Γ, versus the ensemble average fluorescence intensity, I_f, upon a) CF488-streptavidin (for t = 215-350 s, see Figure 2c in the main text) and b) CF488-antibiotin (for t = 160-450 s, see Figure 2d in the main text) binding to the vesicles. The fluorescence signal was corrected for intensity decrease (see Figure 2 in main text) due to quenching and photobleaching. Γ was calculated from the temporal variation in the scattering intensity (rolling 50 s median value of raw intensity) for each individual vesicle, and converted to an equivalent increase in protein layer thickness Δd versus time using a fix n_p of 1.6 (see Eq. 6 in the main text). The mean protein layer thickness of the particles (excluding top and bot 5% outlier values) for each time was then converted to mass using Eq. 6 (black). Essentially identical curves were obtained if the protein layer thickness was instead kept fix at 5 and 15 nm (green) for SA and anti-biotin, respectively, in which case Γ was instead estimated from the increase in the effective refractive of the protein film (see Eq. 6 in the main text).

5. Diffusion-limited binding kinetics

The present experimental setup for the waveguide microscopy measurements involves pipetting a small volume of sample solution into a droplet of buffer solution located on the sensor surface, rapid mixing using the pipette and observing the response at the sensor surface as the sample permeates the droplet. The timescale characterizing relaxation of the solution motion after mixing is rather short (< 1 s as estimated according to hydrodynamics), much shorter that that characterizing the adsorption kinetics, and accordingly the adsorption can be considered to occur under stagnant conditions. To gain further insights regarding the kinetics of the protein adsorption process, we here discuss protein diffusion in this droplet environment.

The used vesicle surface coverage results in approximately 8×10^5 vesicles present on the 4 mm^2 sensor surface. The mean vesicle radius of 52.5 nm results in a maximum (54%) coverage of approximately 935 or 535 proteins/vesicle for SA or antibiotin, respectively. These maximum coverages correspond to ~1% and ~3% of all available material in a 70 µl droplet of 1 µg/mL protein concentration. This indicates a globally diffusion limited binding process, assuming that the protein binding is not governed by local diffusion limitations (i.e. diffusion on distances comparable to the vesicle size). To examine this assumption, we approximate the protein diffusion flux towards a vesicle using the conventional Smoluchowski expression for bulk diffusion:

$$w = 4\pi D R c_{\mathrm{loc}} \tag{S3}$$

where R is the vesicle radius, $D = k_{\mathrm{B}}T/6\pi\eta\rho$ the protein diffusion coefficient (with ρ being the protein hydrodynamic radius, η the solution viscosity) and c_{loc} the local protein concentration (approximated as the global average concentration, 1 µg/mL). This results in a protein diffusion flux to a vesicle on the order of 900 proteins/second, a value considerably larger than what is experimentally observed. This excludes that c_{loc} is close to the average concentration and thus confirms a global diffusion limitation (i.e. on large distances compared to the vesicle size). Given a globally limited diffusion process, the protein flux towards the sensor surface can be approximated as:

$$W = D c_{\infty}/l \tag{S4}$$

where c_{∞} is the bulk protein concentration (~1 µg/mL) and l is the length scale characterizing the diffusion front, which in our case is estimated to be comparable with droplet height above the

sensor (~2-3 mm), resulting in a few proteins per second and vesicle, a value in good agreement with the observations.

References

[1] C. Rosano, P. Arosio, and M. Bolognesi, "The X-ray three-dimensional structure of avidin," *Biomol. Eng.*, vol. 16, no. 1, pp. 5–12, 1999.

[2] B. Agnarsson, M. Mapar, M. Sjöberg, M. Alizadehheidari, and F. Höök, "Low-temperature fabrication and characterization of a symmetric hybrid organic–inorganic slab waveguide for evanescent light microscopy," *Nano Futur.*, vol. 2, no. 2, 2018.

[3] H. Foroosh, J. B. Zerubia, and M. Berthod, "Extension of phase correlation to subpixel registration," *IEEE Trans. Image Process.*, vol. 11, no. 3, pp. 188–199, 2002.

[4] D. L. M. Rupert *et al.*, "Dual-Wavelength Surface Plasmon Resonance for Determining the Size and Concentration of Sub-Populations of Extracellular Vesicles," *Anal. Chem.*, vol. 88, no. 20, pp. 9980–9988, Oct. 2016.

[5] G. Emilsson, R. L. Schoch, L. Feuz, F. Höök, R. Y. H. Lim, and A. B. Dahlin, "Strongly stretched protein resistant poly(ethylene glycol) brushes prepared by grafting-to," *ACS Appl. Mater. Interfaces*, vol. 7, no. 14, pp. 7505–7515, 2015.

[6] B. G. E. Perlmann, "The Specific Refractive Increment of Some Purified Proteins," vol. Ill, no. 1945, 1948.

[7] J. Lokajová, J. Laine, E. Puukilainen, M. Ritala, J. M. Holopainen, and S. K. Wiedmer, "Liposomes for entrapping local anesthetics: A liposome electrokinetic chromatographic study," *Electrophoresis*, vol. 31, no. 9, pp. 1540–1549, 2010.

[8] M. J. Sanderson, I. Smith, I. Parker, and M. D. Bootman, "Fluorescence microscopy," *Cold Spring Harb. Protoc.*, vol. 2014, no. 10, pp. 1042–1065, 2014.
